# How I do it—tubular trans-spinous process muscle-sparing approach for bilateral lumbar microdiscectomy: surgical technique with illustrative video

**DOI:** 10.1007/s00701-025-06648-8

**Published:** 2025-08-22

**Authors:** Anthony M. T. Chau, Aaron Lerch

**Affiliations:** 1 Brisbane Clinical Neuroscience Centre, Mater Hospital Brisbane, South Brisbane, Queensland Australia; 2 Department of Medicine, Griffith University, Gold Coast, Queensland Australia; 3 Department of Medicine, University of Queensland, Herston, Queensland Australia

**Keywords:** Spinous process splitting, Tubular microdiscectomy, Laminectomy, Minimally invasive spinal surgery

## Abstract

This video demonstrates our surgical approach for an entirely tubular trans-spinous process bilateral lumbar discectomy. A 47-year-old man presented with 12 months of severe predominantly right L5 radiculopathy. Magnetic resonance imaging revealed a large diffuse disc bulge at L4/5 resulting in bilateral lateral recess and central stenosis. He underwent bilateral discectomies via a trans-spinous process tubular approach. Postoperatively, his pain resolved and was discharged home the following day. Clinical resolution was sustained at 6 weeks. An entirely tubular trans-spinous process technique for lumbar decompression provides excellent bilateral lateral recess exposure through a minimally invasive muscle-sparing approach.

**Supplementary Information:**

The online version contains supplementary material available at 10.1007/s00701-025-06648-8.

## Relevant surgical anatomy

The lumbar spinous processes are typically broad and more horizontally-oriented when compared to their cervical and thoracic counterparts [1]. They provide attachment points for the erector spinae and transversospinalis muscles, and are supported by the supraspinous and interspinous ligaments [1]. The spinous process connects with the lamina in the midline. On the inner surface of the lamina attaches the deep layers of the cephalad and caudal ligamentum flava [3]. Between these attachment points of these ligamentum flava exists a deficiency, where a thin layer of only epidural fat separates the mid-region of the lumbar lamina from the dura [3].

A variety of decompressive techniques have evolved for the management of degenerative lumbar spinal canal stenosis [6]. Conventional open laminectomy has been criticised for its disruption of the paraspinal muscles, alongside excess bony disruption that contributes to postoperative pain, excessive bleeding, and prolonged recovery [4, 9]. The rise of minimally invasive surgical approaches aims to reduce this disruption whilst still achieving excellent decompression, with tubular and endoscopic techniques shown to have superior outcomes for intraoperative bleeding, postoperative pain, length of stay, and reoperation rates compared to open approaches [4, 9].

Most tubular techniques approach the lumbar spine unilaterally to achieve a bilateral decompression, thereby remaining lateral to the spinous process and still introducing some paraspinal muscle disruption. Some authors have proposed that this disruption can be minimised via a spinous-splitting approach, whereby the spinous process is divided into two halves longitudinally, providing a central access corridor without detaching the paravertebral muscles [2, 5, 7, 8]. This technique traditionally has been via an open approach, although some authors have inserted tubular retractors after the initial open spinous process split [8]. However to our knowledge, there has been no description of performing this approach entirely through a tube.

Secondly and more significantly, a trans-spinous process compared to a standard unilateral approach is a pure midline approach. This, therefore, affords superior simultaneous visualisation of both lateral recesses when this is required. Whilst the contralateral ligamentum flavum can be easily removed via the standard unilateral approach, a contralateral discectomy can be more challenging and involve significant dural retraction. In such a situation, bilateral exposures are necessary, increasing muscle disruption and operative time.

## Description of the technique

**Fig. 1:**
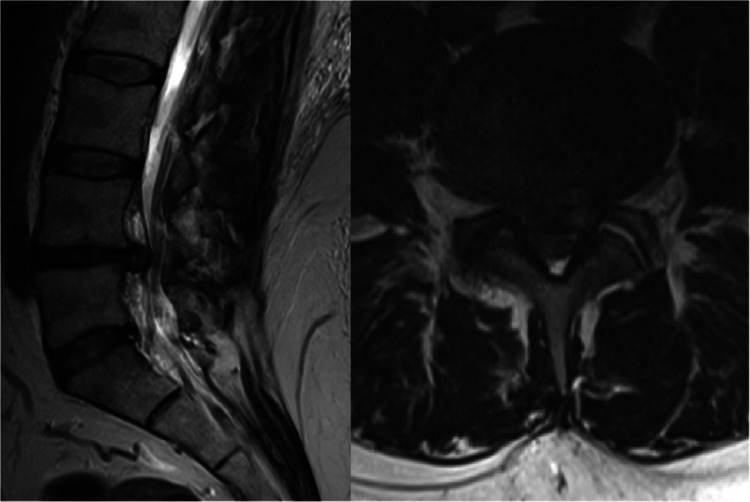
Preoperative sagittal and axial T2 MRI demonstrating a diffuse central disc bulge causing severe spinal canal stenosis with bilateral L5 nerve root compression

A 47-year-old male (body mass index 42kg/m^2^) presented with 12 months of severe L5 radiculopathy, much worse on the right. Neurological examination was normal for lower limb power and reflexes. Magnetic Resonance Imaging (MRI) demonstrated severe spinal canal stenosis due to a large central diffuse disc bulge with bilateral lateral recess stenosis (Fig. 1). MRI revealed the distance from the patient’s skin to the dura at L4/5 to be 9cm. After a trial of conservative therapy, including a right L5 cortisone block, the patient decided to proceed to surgical decompression.

The following is demonstrated in our appended 3-min surgical video (Video 1). Under general anaesthesia, the patient was placed prone on a Jackson table. A lateral x-ray was performed to mark the L4/5 level. A 2 cm vertical midline skin incision was made, and a 5 cm long, 16 mm wide Metrix tube (Medtronic) was inserted onto the tip of the L4 spinous process. X-ray confirmed the trajectory remained towards the L4/5 disc level. The operating microscope was introduced. Monopolar cautery was used to remove the overlying supraspinous ligament and define the bony anatomy (we use a long guarded monopolar tip, which we bend into a bayoneted shape). A 2.5mm angled matchstick drill used in conjunction with an 8 French sucker was then used to drill through the cancellous bone of the spinous process. Whilst the superficial cortical bone of the spinous process was breached craniocaudally, the spinous process was only shelled out laterally so as not to disrupt paravertebral muscle attachments and incur unnecessary bleeding.

Drilling proceeded meticulously and cautiously down the narrow cancellous corridor until deep cortical bone was encountered. Care was taken at this point to leave a shell of cortical bone overlying the epidural space to later allow for tubular dilators to safely dock. The microscope and drill were then angled slightly more proximally to detach the cortical bone at each spinolaminar junction craniocaudally. The smallest tubular dilator was then placed under direct vision down the surgical corridor, resting on the residual bone of the spinolaminar junction. The 5 cm tubular retractor was withdrawn, and then serial dilators were placed. A 7 cm long 16 mm wide tubular retractor was inserted via the trans-spinous process pathway with lateral x-ray to confirm appropriate angulation and depth. It is not necessary to dock this longer tube all the way down to the spinolaminar junction, rather only a tube of sufficient depth to keep the paravertebral muscles out of the surgical field is required.

Under the microscope, a routine bilateral laminotomy and undercutting of the medial facets was performed to skeletonize the ligamentum flavum and its attachments craniocaudally. A dissector was used to reveal the epidural fat and dura, and Kerrison rongeurs were used to resect the flavum and undercut the medial facet joints. Dissection was performed to mobilise the L5 nerve roots, and bilateral discectomies were performed sequentially to remove tough fibrous disc bulge, ensuring the discectomy met at the midline with resection of displaced posterior longitudinal ligament and herniated disc material. An angled ball probe was used to confirm complete neural decompression bilaterally. Hemostasis was achieved. The tubular retractor was carefully withdrawn, cauterising any bleeding points. Three interrupted vicryl sutures were placed in the dermis, and dermabond glue applied to the skin. Estimated blood loss was 20 ml, and the operating time was 120 min (longer than a standard case due to obesity).

**Fig. 2:**
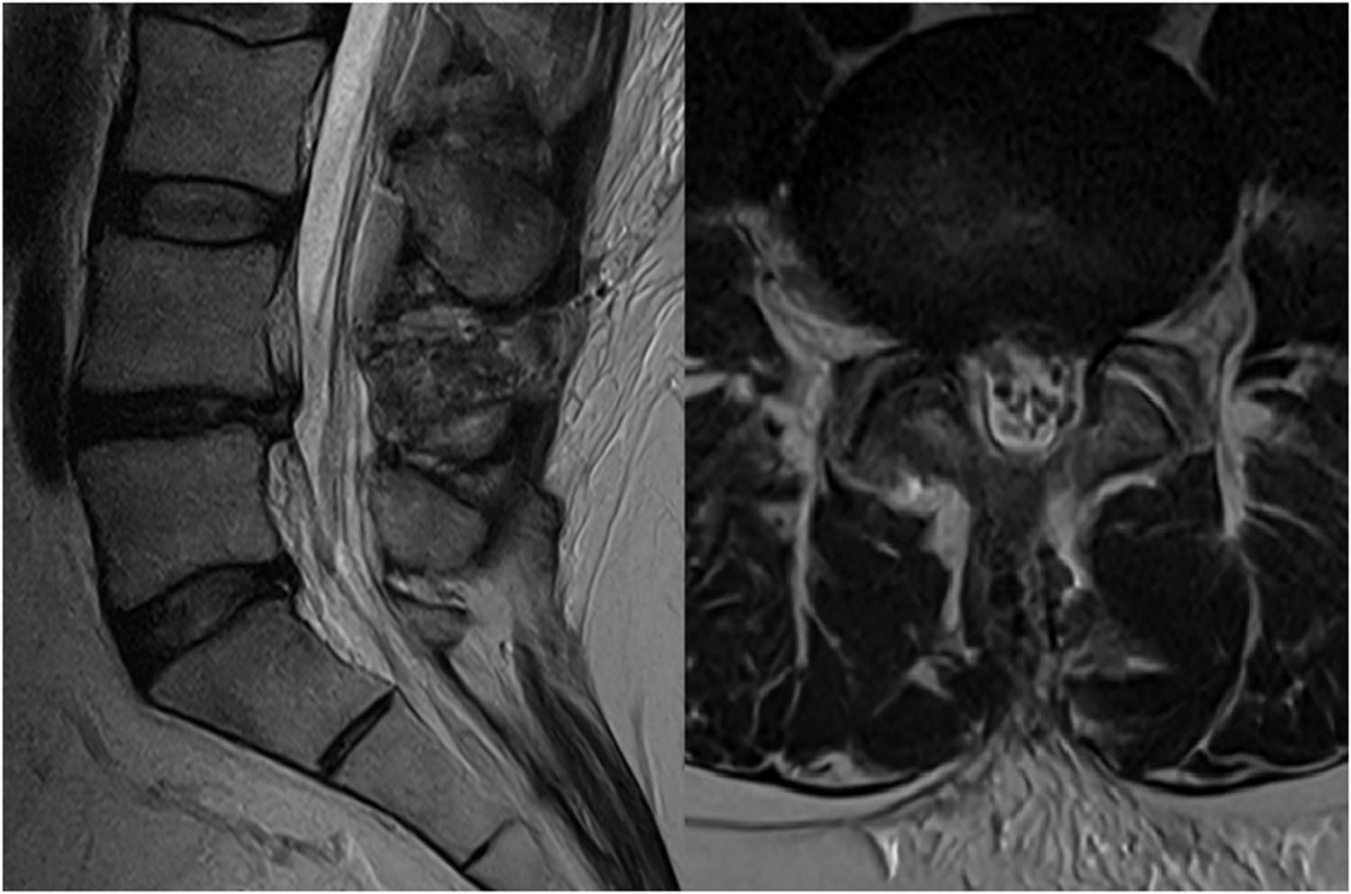
Day 1 postoperative sagittal and axial T2 MRI demonstrating complete decompression of neural elements with no paravertebral muscle collateral injury. The sagittal view illustrates the trans-spinous process corridor taken during the operation

The patient reported complete resolution of his L5 radiculopathy, with negligible back pain postoperatively. Day 1 postoperative MRI demonstrated complete decompression of the spinal canal (Fig. 2). He was discharged home the day after surgery with no oral analgesic requirement. At 6 weeks follow-up, his symptom resolution was sustained, and he was discharged from further routine follow-up.

## Indications

Trans-spinous approaches (spinous process splitting) are used commonly in open laminectomies due to the benefits of sparing paravertebral muscle dissection and postoperative denervation/atrophy, although disrupts the supraspinous ligament and spinous process. The surgical technique described in this case report adapts the spinous process splitting approach for minimally invasive tubular decompressions. By performing the initial spinous process split under the microscope using a shorter initial tubular retractor, the entire operation can be performed using only a 2 cm skin incision.

However, we propose that the true benefit of this pure midline approach lies in the superior visualisation of the spinal canal when bilateral discectomies are required, compared to the standard unilateral tubular approaches. In a case such as that presented here, bilateral tubular approaches would normally be required to adequately decompress bilateral large firm disc bulges, which adds significantly to operating time. Because of this, some tubular spinal surgeons may opt for a standard open approach for such a case. Thus, employing a trans-spinous process tubular technique harnesses the benefits of both minimally invasive tubular surgery with minimal collateral tissue damage, and an excellent midline exposure with bilateral lateral recess access when it is required.

## Limitations

We would rate the difficulty of this minimally invasive pure midline tubular approach as only marginally higher than that of a standard tubular microdiscectomy. However, the anatomy of the spinous process should be carefully analysed on the preoperative MRI, particularly in relation to its sagittal location relative to the disc space, any lateral deviation, and its width (surgical corridor). A narrow spinous process naturally increases the difficulty of access. In general though, we have found that a standard angled 2.5mm matchstick drill and 8 French suction device is suitable.

In cases where the spinous process is felt to be too thin to allow for the above corridor, we have also modified this technique successfully using a tubular bilateral subperiosteal monopolar exposure, followed by focal spinous process resection using a pituitary rongeur and/or drill. This latter modification also leads to very minimal muscle disruption, is slightly faster, and surgery is similarly completed through a 2 cm incision.

### How to avoid complications

1. Carefully analyse the preoperative MRI to plan the surgical corridor.
2. Pay particular attention during drilling once cancellous bone has been removed and the deep cortical bone of the inner lamina has been reached. Beneath this, there may be a deficiency of the ligamentum flavum, and only a thin layer of epidural fat overlying the dura.
3. Leave a shelf of inner lamina cortical bone to protect the dura and to avoid plunging whilst inserting the tubular dilators.
4. Use intraoperative x-ray to confirm level, angulation and depth.

### Specific information for the patient

Combining the muscle-sparing techniques of tubular microsurgery and spinous-process splitting allows excellent minimally invasive bilateral lumbar lateral recess access for bilateral discectomies.

## Supplementary Information

Below is the link to the electronic supplementary material.

Supplementary file1 (MP4 249502 KB)

## Data Availability

No datasets were generated or analysed during the current study.
